# Observational Study: Preliminary Evaluation of Nostril Narrowing in Relation to Unilateral Mastication

**DOI:** 10.3390/jcm14041319

**Published:** 2025-02-17

**Authors:** Miguel Ramón Pecci-Lloret, Carmen María García-Sampedro, Julia Guerrero-Gironés, Emilio López-Jiménez

**Affiliations:** 1 Gerodontologý an Special Care Dentistry Unit, Morales Meseguer Hospital, Faculty of Medicine, University of Murcia, 30008 Murcia, Spain; miguelramon.pecci@um.es (M.R.P.-L.); carmengarsam@gmail.com (C.M.G.-S.); 2 Private Practice, C. Angosto, 55, 23280 Beas de Segura, Spain; ddnariz@gmail.com

**Keywords:** unilateral mastication, nasal asymmetry, craniofacial anatomy, chewing habits

## Abstract

**Background:** Unilateral mastication (UM), defined as the habitual use of one side of the mouth for chewing, has been linked to various craniofacial asymmetries and systemic effects. This study aims to explore the relationship between UM and nasal airway asymmetry, investigating whether a habitual chewing side correlates with a narrowed nostril. These findings provide a preliminary understanding of how chewing practices might impact craniofacial anatomy and respiratory health. **Methods:** A pilot study involving 24 adults aged 18 and older was conducted. Inclusion criteria excluded individuals with prior orthodontic treatment or edentulism. The habitual chewing side was determined through self-report and direct observation of chewing gum activity. Nostril dimensions were assessed using ImageJ^®^ 1.54 software by blinded observers. Statistical analysis, including Fisher’s exact test and Kruskal-Wallis test, was performed using R version 4.4.1 to examine associations between the chewing side and nasal asymmetry. **Results:** Half of the participants (50%) preferred the right side for chewing. A significant association was found between the chewing side and the smaller nostril (*p* < 0.001). Participants with a smaller right nostril consistently preferred chewing on the right side, with a large effect size (ϕ = 0.845). No significant differences in age were observed across nostril groups (*p* = 0.47). **Conclusions:** This study reveals a strong association between unilateral mastication and nasal airway asymmetry, highlighting the potential role of chewing habits in influencing craniofacial development. These findings emphasize the need for further longitudinal studies to explore the systemic and aesthetic implications of habitual chewing patterns.

## 1. Introduction

Chewing is a complex process, in which many elements of the stomatognathic system intervene. Unilateral mastication (UM) refers to the habitual use of one side of the mouth during chewing, a pattern observed in a significant portion of the population. This behavior has garnered attention due to its potential to induce various asymmetries in craniofacial structures, leading to health concerns such as temporomandibular disorders (TMD), occlusal imbalances, and even systemic effects like hearing loss [1,2,3]. UM has been extensively associated with altered functional and postural dynamics of the masticatory system, which may further impact structures related to the airway and facial symmetry [4,5,6].

Research on the impact of unilateral mastication has predominantly focused on the temporomandibular joint (TMJ) and occlusal forces, demonstrating that these repetitive habits can lead to an overload on one side of the dental arches. This imbalance not only affects dental and joint health but can also contribute to significant skeletal changes, including development of the maxilla [4,7]. However, less is known about how this habitual asymmetry in chewing might affect the anatomy of the nasal airways, specifically regarding the potential narrowing of the nasal passage on the preferred chewing side.

In addition to the previously described associations between unilateral mastication (UM) and craniofacial structures, recent research suggests that masticatory patterns may also influence postural balance and spinal alignment. A study highlights a potential relationship between altered mastication and the development of scoliosis, emphasizing the interconnectedness between the stomatognathic system and the musculoskeletal system [8,9,10].

These findings strengthen the hypothesis that mastication not only affects localized structures but may also have systemic impacts on anatomically and functionally related areas. Similarly, the interaction between masticatory patterns and upper airway structures, such as the nostril, might be mediated by muscular mechanisms or adaptive anatomical changes. This perspective underscores the clinical relevance of this study and suggests future research avenues into the systemic implications of masticatory habits.

The nasal airway plays a crucial role in respiration, and its structure can be sensitive to external forces and functional patterns, including those originating from the masticatory system [11]. Studies on craniofacial growth in both human and animal models suggest that altered masticatory function can influence the development of nasal and sinus structures. In particular, unilateral masticatory function has been linked to changes in the maxillary sinuses, the glenoid fossa, and potentially the nasal airway, which may contribute to breathing difficulties and other respiratory issues [12,13].

Despite these observations, there is scant research regarding the direct relationship between unilateral mastication and nasal airway narrowing. This represents an important lacuna in literature since any conceivable change in nasal anatomy might spill over to relevance in airway management and craniofacial treatment planning as well. Nasal airway narrowing impairs breathing patterns, which in turn exacerbates other conditions such as sleep-disordered breathing and increased oral respiration [1,2]. Furthermore, asymmetry in the nostril may also result in aesthetic issues, affecting the appearance of the nose and the overall harmony of the face [14].

Since it is a pilot study, it is planned to find out whether unilateral mastication is associated with the preferred chewing side by narrowing the nasal airway. For this reason, this study investigates 24 patients and describes preliminary data about the influence of habitual chewing practices on nasal anatomy by providing a basis for further studies in the effects of masticatory habits on craniofacial development and respiratory health in general.

This study aims to investigate the potential relationship between unilateral habitual chewing and nostril asymmetry. Specifically, we seek to determine whether a significant correlation exists between the preferred chewing side and nostril size, offering preliminary data to guide future studies on the effects of masticatory habits on craniofacial development and respiratory health.

## 2. Materials and Methods

This study was reviewed and approved by the Ethics Committee of the University of Murcia (approval No. M10/2024/097). Its design adhered to the STROBE (Strengthening the Reporting of Observational Studies in Epidemiology) guidelines for cross-sectional studies [15].

### 2.1. Patient Selection

The inclusion criteria were as follows, ensuring homogeneity of the study sample:

- Adults aged 18 years or older.
- Subjects that have never received any orthodontic treatment of any kind and are not edentulous.
- participants gave informed consent before being included in this study.

This study was conducted at the University Dental Clinic of the University of Murcia between January and September 2024. Recruitment, exposure, follow-up, and data collection were carried out during this period.

### 2.2. Determination of Chewing Side

The habitual chewing side was determined by two major methods:

1. Preferential Chewing Side: This was self-reported by the subjects in the first visit and one week later to check for reliability in the response.
2. Observational Method: Patients are given a piece of chewing gum and are asked to chew for a minute. The predominant chewing side is observed by a trained clinician.

### 2.3. Data Collection

Following are the details of everything done to collect comprehensive data.

- Intraoral Photography: Photographs were taken in maximal intercuspation to document the dental arches in order to visualize and determines the chewing side.
- Orthopantomography: For panoramic radiographs, the mandibular and dental structures were checked.
- Nostril Photography: Photography at the shooting details of the nostril was carried out for the analysis of general features and dimensions of the nose.

### 2.4. Independent Observer Evaluation

**Figure 1:**
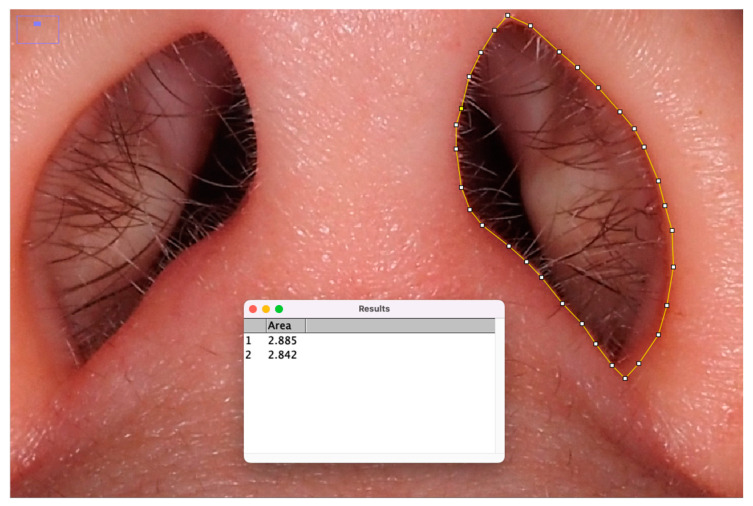
Example of how nostril was measured by the observers. The right and left nasal apertures were outlined using ImageJ^®^ 1.54 software, and the areas of each nostril were calculated in square units. Observers were blinded to the patients’ reported chewing side during this measurement process to ensure unbiased analysis.

Two blinded observers, other than the original examiner, analyzed the obtained images without prior information regarding the patient’s reported chewing side. The two observers, using ImageJ^®^ 1.54 software, measured the openings of both the right and left nostril and noted which was smaller (Figure 1).

### 2.5. Statistical Analysis

Statistical analyses were conducted using R version 4.4.1 [16].

The following tests were performed:

- Descriptive Analysis: Median, mean, and standard deviation were calculated for numerical variables, and frequencies for categorical variables.
- Normality Tests: Shapiro-Wilk test was applied to determine the normality of numerical variables.
- Association Analysis:
  ○ Fisher’s exact test was used to analyze the relationship between the chewing side and the smaller nostril.
  ○ Kruskal-Wallis test was applied for non-parametric comparisons of age across groups.
- Significance Level: Results were considered significant if *p* < 0.05.

## 3. Results

The study included 24 participants, of whom 62.5% (*n* = 15) were female and 37.5% (*n* = 9) were male.

Initially, 30 individuals were assessed for eligibility. Five individuals did not participate: three declined to sign the informed consent, and three refused to be photographed after reading the participant information sheet and, therefore, did not provide consent. No missing data were recorded for any of the variables of interest among the final 24 participants.

**Figure 2:**
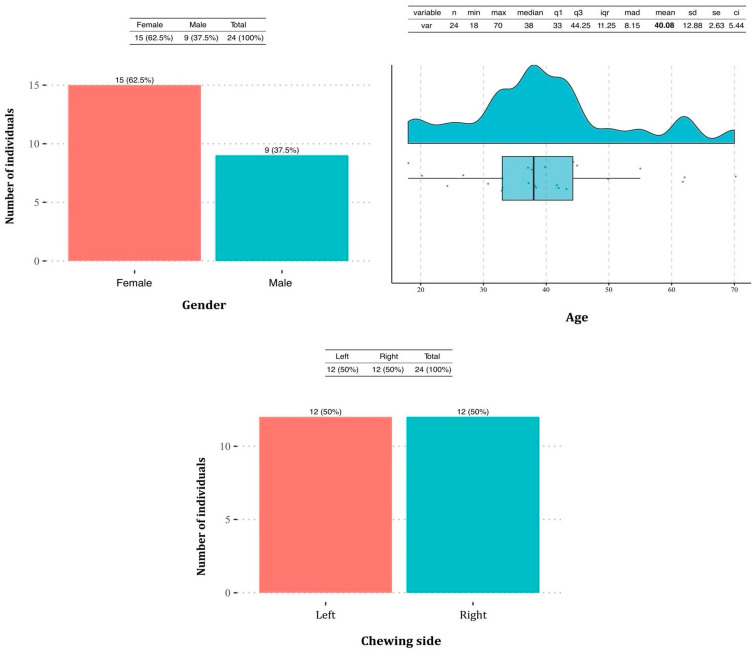
Descriptive analysis of the study population. The first graph illustrates the distribution of participants by gender, showing 62.5% females (*n* = 15) and 37.5% males (*n* = 9). The second graph represents the age distribution, with a median of 38 years and an interquartile range of 33 to 44 years. The third graph displays the distribution of the preferred chewing side, with an equal distribution of participants favoring the left (50%, *n* = 12) and right (50%, *n* = 12) sides.

The median age of the participants was 38 years, ranging from 18 to 70 years. Regarding chewing side preferences, the distribution was perfectly balanced, with 50% (*n* = 12) of the participants favoring the right side and the other 50% (*n* = 12) favoring the left side (Figure 2).

**Figure 3:**
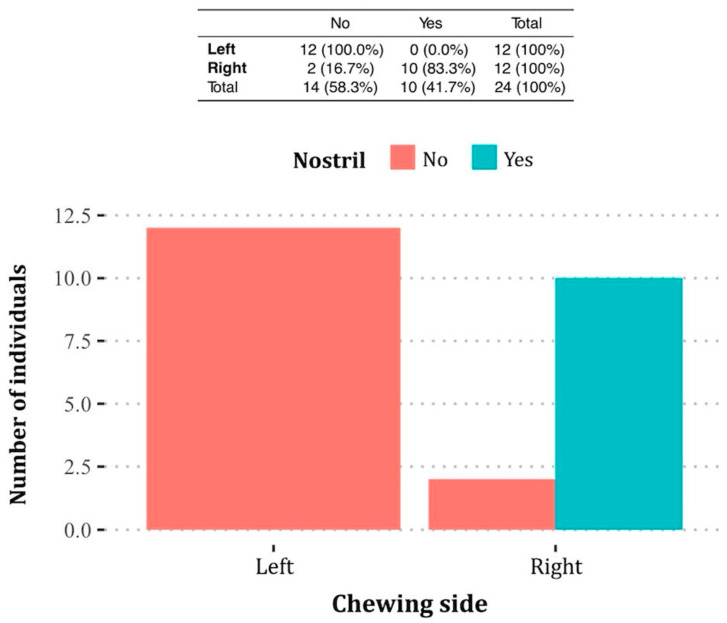
Contingency table and bar graph showing the relationship between the preferred chewing side (left or right) and nasal window asymmetry. “Yes” indicates the presence of a smaller right nostril, while “No” indicates a smaller left nostril.

**Figure 4:**
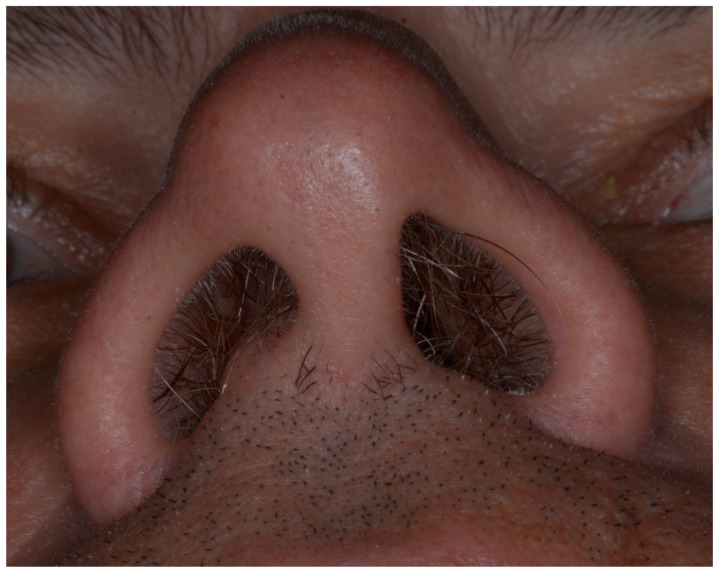
Example of a right-side chewer with a visibly smaller and narrower right nostril compared to the left. This illustrates the observed association between the preferred chewing side and nasal asymmetry, as documented in the study findings.

An analysis of nostril asymmetry revealed that 41.7% (*n* = 10) of participants exhibited a smaller right nostril, while 58.3% (*n* = 14) had a smaller left nostril. A significant association was observed between the preferred chewing side and the smaller nostril (*p* < 0.001) (Figure 3). Specifically, participants who chewed predominantly on the right side were more likely to have a smaller right nostril. This relationship is illustrated in Figure 4, which provides an example of a right-side chewer with a visibly narrower right nostril, reinforcing the observed link between chewing side and nasal asymmetry.

These findings suggest a strong association between unilateral chewing habits and nasal airway asymmetry, providing novel insights into the potential anatomical and functional implications of habitual masticatory patterns.

### Effect Size

The phi coefficient for the relationship between chewing side and nostril was ϕ = 0.845, indicating a large effect size. This suggests a strong association between these variables.

According to the table, all participants who prefer chewing on the left side (*n* = 12) have a smaller left nostril (“No” category). In contrast, 83.3% (*n* = 10) of those who prefer chewing on the right side have a smaller right nostril (“Yes” category). The bar graph visually represents this distribution, highlighting a significant association between the chewing side and nostril asymmetry (*p* < 0.001).

## 4. Discussion

The aim of the present study was to investigate the relationship between unilateral habitual chewing and asymmetry of nasal apertures in patients in an adult population. Our findings showed a significant statistical relationship existed regarding the selected side of chewing and the smaller nostril. Notably, subjects with a smaller right nostril showed preference for chewing on the right side, which was reflected in the strong effect size: ϕ = 0.845. These findings suggest that a functional and, possibly, anatomic relation exists between the asymmetry of the nasal airway and habitual chewing.

However, nasal asymmetry may not always be exclusively related to masticatory patterns. Anatomical factors such as nasal septum deviation or previous nasal trauma, which are common in the general population, could also contribute to nostril size differences [17]. Additionally, unilateral mastication might not only influence nostril asymmetry but could also potentially be associated with nasal septum deviation. To confirm this hypothesis, future studies should incorporate CT scans to evaluate nasal septum deviation and its possible relationship with masticatory habits.

### 4.1. Comparison with the Existing Literature

Previous studies demonstrated that unilateral mastication alters the development of craniofacial structures such as the TMJ, maxillary sinuses, and mandibular development [1,6]. However, the clear explanation of the analysis concerning asymmetry in the nostril and its relationship with chewing pattern was not fully explained. Our results agree with those investigations that stated, because of habitual continuous function like unilateral mastication, asymmetry in supra-adjacent structures might be caused or enhanced, This phenomenon is observed not only in dentistry but also in other fields, such as sports medicine, where repetitive functional habits contribute to asymmetry and structural changes [18,19].

Furthermore, recent studies have highlighted a potential link between unilateral mastication and postural imbalances, including scoliosis [8,20]. This suggests that masticatory function may influence musculoskeletal systems beyond the craniofacial region. These observations align with our hypothesis that chewing patterns may impact the nasal airway and related anatomical features, emphasizing the interconnected nature of craniofacial and postural systems.

### 4.2. Potential Mechanisms

This might be explained by the fact that structures in the craniofacial complex are developmentally and functionally interrelated, and unilateral masticatory forces can result in either a mechanical influence on the pattern of tissue remodeling of the nasal and maxillary tissues or a change in airflow dynamics [21]. The hypertrophy of the masticatory muscles acting on the side of preferred chewing also may be another reason for the asymmetry of the anatomical spare side features [12,22].

Furthermore, the link between mastication and nasal airway asymmetry may also involve neuromuscular adaptations. Research suggests that prolonged unilateral activity can alter the coordination of muscles involved in mastication and adjacent structures, potentially leading to compensatory changes in other areas, such as the nasal and pharyngeal regions [23]. These findings highlight the complexity of interactions between functional habits and anatomical structures, providing new insights into the broader implications of habitual behaviors.

### 4.3. Clinical Implications

Understanding how mastication and nasal asymmetry are related will have overwhelming relevance to both dentistry and otolaryngology. Identifying habitual chewing patterns as one of the possible causes of nasal airway asymmetry may probably allow for an improved diagnosis and management of the related pathologies such as nasal obstruction or temporomandibular disorders. In addition, such findings emphasize the importance of encouraging balanced mastication as a mode of prevention or reduction in asymmetric craniofacial development.

In addition to its functional impact, nasal airway asymmetry can also have aesthetic consequences, affecting facial harmony and patient self-esteem. Studies have shown that individuals with nasal deformities, such as a crooked nose, often experience reduced self-esteem and quality of life. Surgical correction of these deformities can lead to significant improvements in both appearance and psychological well-being [24].

### 4.4. Limitations

While the findings are intriguing, there are a few limitations surrounding this study: First, the relatively small sample size, comprising 24 participants, limits generalizability of the findings, as any observed associations would need to be confirmed in a much larger cohort representative of a more diverse population.

Second, the absence of imaging studies such as CT scans means that nasal septum deviation, which could influence nostril dimensions, was not evaluated. This limits the ability to fully distinguish the effects of masticatory habits from underlying anatomical variations.

The cross-sectional design of the research does not allow determining the cause-effect relationship between habitual chewing patterns and nasal asymmetry. It is unknown whether the asymmetry results from the chewing habits or if it predisposes the individuals to the use of one side of the mouth.

Third, though ImageJ 1.54 software permits the accurate measurement of nasal dimensions, manual input of data ensures observer bias during this process is inevitable, despite the fact that the evaluators were blinded to the patients’ reported chewing sides. Some limitations are mentioned herein; thus, further studies need to build up from these findings with better methodologies and increased sample sizes.

Further research should focus on:

Longitudinal studies: By following the patients in time, it would detect if unilateral chewing habits produce progressive nasal asymmetry.

Larger Sample Sizes: To ensure increased statistical power and thus confirm these results in heterogeneous populations.

Intervention Studies: These will determine if interventions aimed at symmetric mastication reduce or reverse nasal asymmetry.

## 5. Conclusions

This study highlights a significant association between habitual chewing patterns and nostril asymmetry, providing novel insights into the interplay between masticatory function and craniofacial anatomy. These findings lay the groundwork for further exploration into the clinical relevance of mastication in the context of nasal and respiratory health, further research with larger sample sizes is necessary to confirm and expand upon these results.

## Data Availability

The original contributions presented in this study are included in the article. Further inquiries can be directed to the corresponding author.
